# Shear-wave elastography of the plantar fascia: a systematic review and meta-analysis

**DOI:** 10.1007/s40477-022-00770-4

**Published:** 2023-01-20

**Authors:** Domenico Albano, Carmelo Messina, Salvatore Gitto, Francesca Serpi, Mariachiara Basile, Marzia Acquasanta, Ezio Lanza, Luca Maria Sconfienza

**Affiliations:** 1 Unità Operativa di Radiologia Diagnostica e Interventistica, IRCCS Istituto Ortopedico Galeazzi, 20161 Milan, Italy; 2 Dipartimento di Scienze Biomediche per la Salute, Università degli Studi di Milano, 20133 Milan, Italy; 3 Scuola di Specializzazione in Radiodiagnostica, Università degli Studi di Milano, 20122 Milan, Italy; 4 Radiologia e Diagnostica per immagini, Ospedale Città di Sesto San Giovanni - ASST Nord Milano, 20099 Sesto San Giovanni, Italy; 5 Department of Radiology, Humanitas Clinical and Research Center – IRCCS, 20089 Rozzano, Italy

**Keywords:** Shear wave elastography, Ultrasound, Velocity, Stiffness, Plantar fascia, Heel pain

## Abstract

**Purpose:**

To assess the efficacy of shear-wave elastography (SWE) of the plantar fascia (PF) in identifying plantar fasciitis.

**Methods:**

A literature search was conducted on the PubMed and Medline databases for articles published up to August 2022. The Newcastle–Ottawa scale was used to assess the risk of bias. We included original research studies in English dealing with the evaluation of patients with plantar fasciitis by means of SWE and including shear modulus (KPa) and/or shear-wave velocity (m/s). We compared healthy and pathologic PF stiffness using the standardised mean difference (SMD) in a random-effects model (95% CI).

**Results:**

Five studies were included with a total of 158 pathologic PFs and 134 healthy PFs. No significant publication bias was detected. Studies were highly heterogeneous (*p* < 0.00001; *I*^2^ = 97%). Pathologic PFs showed significantly lower stiffness, with an SMD of − 3.00 m/s (95% confidence interval: − 4.95 to − 1.06, *p* = 0.002), compared to healthy PF.

**Conclusion:**

Pathologic PFs present significantly lower stiffness than healthy PFs. However, the analysed studies are highly heterogeneous.

## Introduction

Plantar fasciitis is a multifactorial disease, mostly determined by mechanical overload and age-related degenerative changes of the plantar fascia (PF) [1, 2]. It is the leading cause of heel pain in adult subjects with a non-negligible impact on patients’ quality of life [3]. Imaging is routinely used to support clinical examination, with plain radiography being able to detect calcaneal spurs and PF calcifications/ossifications, while ultrasound is applied to identify the increased thickness, hypoechoic texture, and any partial/full-thickness tears [4]. Ultrasound is also used as guidance to increase the accuracy and effectiveness of interventional procedures to treat plantar fasciitis [5].

Strain elastography enables us to evaluate non-invasively the mechanical properties of biologic tissues, specifically tissue stiffness, by assessing the displacement of soft tissue structures after external compression [6]. It is increasingly used in musculoskeletal settings, already having an established role in lateral epicondylitis and Achilles tendinopathy [4]. Previous studies have shown that it might be used to highlight the softer appearance of the PF in plantar fasciitis, increasing the diagnostic performance of B-mode ultrasound and being supportive in doubtful cases with inconclusive ultrasound findings [7]. Some of the drawbacks of this technique, including the limited reproducibility and the qualitative nature of data, have led to the introduction of shear-wave elastography (SWE) as a quantitative and more objective tool to estimate soft tissues’ stiffness by using an acoustic radiation force pulse sequence to produce shear waves that propagate perpendicularly from the transducer with transient structures displacements [6]. Although SWE was investigated first in other districts (i.e. breast imaging), several research lines have been carried out to understand the actual role and additional value of SWE in musculoskeletal imaging. As a matter of fact, some interesting papers have been published about the use of SWE to evaluate the PF over the last few years.

The purpose of this systematic review and meta-analysis was to evaluate the technical performance of SWE in measuring PF stiffness under healthy and pathological conditions.

## Methods

### Literature search strategy

Local Ethics Committee approval was not needed because of the nature of this study, which was a systematic review and meta-analysis. An electronic literature search was conducted on the PubMed and Medline databases for articles published up to August 2022. The databases were evaluated based on the following algorithms: (elastography OR elastogram OR shear wave) AND (plantar fasciitis OR plantar fasciopathy OR heel pain). Studies were first screened by title and abstract, and then the full text of eligible studies was retrieved for further review. The references of identified publications were checked for additional publications to include. The literature search and study selection were performed by one reviewer and double-checked by another independent reviewer. The Newcastle–Ottawa scale was used to assess the risk of bias.

### Inclusion and exclusion criteria

The inclusion criteria were (i) original research studies dealing with the evaluation of healthy PFs or plantar fasciopathy by means of SWE; (ii) involvement of human participants; (iii) English language; (iv) statement that approval from the local ethics committee and informed consent from each patient or a waiver for it was obtained.

The exclusion criteria were (i) studies reporting insufficient data for outcomes or overlapping patient cohorts; (ii) studies using other elastography modalities, such as strain elastography; (iii) case reports and case series involving fewer than ten patients; and (iv) narrative reviews, guidelines, consensus statements, editorials, letters, comments, or conference abstracts.

### Data extraction

Data regarding the following parameters were extracted using a standardised form and analysed:

i. study characteristics: first author, year of publication, and study design;
ii. population characteristics: number of patients and controls, average age, sex, and PF status (healthy vs pathological);
iii. measurement methods: scanning protocol; type of exercise or intervention, if any;
iv. study outcomes:
  - shear modulus (KPa) and shear-wave velocity (m/s) at baseline;
  - shear modulus (KPa) and shear-wave velocity (m/s) after exercise;
  - shear modulus (KPa) and shear-wave velocity (m/s) after intervention;
  - inter-observer reproducibility of measurements performed by different observers, calculated as intraclass correlation coefficient (ICC);
  - intra-observer reproducibility of measurements performed by the same observer in different sessions (ICC).

The meta-analysis was performed using a random-effects model for continuous data (Review Manager (RevMan) [Computer program]. Version 5.4. The Cochrane Collaboration, 2020.), considering the standardised mean difference.

## Results

### Study selection

A total of 29 studies were considered eligible after the literature search. We excluded nine studies that did not report SWE values of stiffness, one study written in Chinese, three studies that did not investigate the PF, and one systematic review. Of the remaining 15 studies, 10 studies were included in our systematic review but excluded from the meta-analysis since 7 studies focused only on healthy PFs, 1 study investigated only pathologic PFs with no comparison with healthy PFs, and the raw data of 2 studies were not available. Hence, 5 studies were finally included in our meta-analysis.

### Studies not included in the meta-analysis

Ten studies that investigated the role of SWE in the evaluation of PFs were included in our systematic review but excluded from the meta-analysis. Chen et al. used SWE to compare the stiffness of healthy runners adopting a rearfoot strike and a forefoot strike [8]. The SWE velocity of forefoot strikers (6.2 ± 0.56 m/s) was significantly lower than that of rearfoot strikers (6.67 ± 0.48 m/s, *p* = 0.01), without any significant difference in terms of echotexture (*p* = 0.54) and PF thickness (*p* = 0.50). Chino et al. found a significant difference in the SWE velocity of healthy subjects between the neutral position (7.8 ± 0.4 m/s) and toe dorsiflexion (9.9 ± 0.3 m/s; *p* = 0.002) in the distal portion of the PF, without significant differences in the insertional portion (5.4 ± 0.6 m/s and 5.5 ± 0.5 m/s, respectively; *p* = 0.88) [9]. Vita et al. did not find statistically significant changes in PF stiffness in healthy users of hormonal contraceptives (*p* > 0.05)[10]. Taz et al. published some studies in healthy subjects reporting no statistically significant differences in the SWE velocity of the PF based on gender (males 6.5 ± 0.7 m/s, females 6.4 ± 0.6 m/s; *p* = 0.673) [11], plantar pressure distribution (mean SWE velocity of 7.7 ± 1.1 m/s; *p* > 0.05) [12], and presence of hallux valgus deformity (mean SWE velocity of 7.6 ± 1.0 m/s vs 7.6 ± 1.0 m/s of controls; *p* = 0.949) [13]. Putz et al. evaluated only patients with plantar fasciitis, observing higher values of stiffness (mean SWE velocity of 5.08 ± 2.24 m/s) in the most painful areas of the PF, but without reporting any statistical results on SWE [14]. Ramu and colleagues compared healthy and pathological PFs, reporting significantly lower SWE velocities in pathologic PFs (*p* < 0.001) using a cut-off value of Young’s modulus of the PF for the diagnosis of plantar fasciitis of ≤ 99.286 kPa (SWE velocity of ≤ 5.75 m/s), with maximum accuracy of 98.3% [15]. Baur et al. found a mean SWE velocity of 6.94 m/s in healthy PFs and of 4.98 m/s in pathologic PFs and mean stiffness of 152.88 kPa and 93.54 kPa, respectively (*p* < 0.001), reaching about 80% specificity and sensitivity using cut-off values of 6.16 m/s for SWE velocity and of 125.57 kPa for stiffness [16]. Shiotani et al. reported significantly higher SWE velocity values of PF in trained runners’ left feet (9.4 ± 1.0 m/s) than in their right feet (8.9 ± 0.9 m/s), while no significant differences were observed in untrained subjects (8.5 ± 1.5 m/s and 8.6 ± 1.7 m/s, respectively), highlighting that stiffer PFs in the left feet of runners may be determined by adaptation related to asymmetrical mechanical loading [17].

### Meta-analysis

**Fig. 1:**
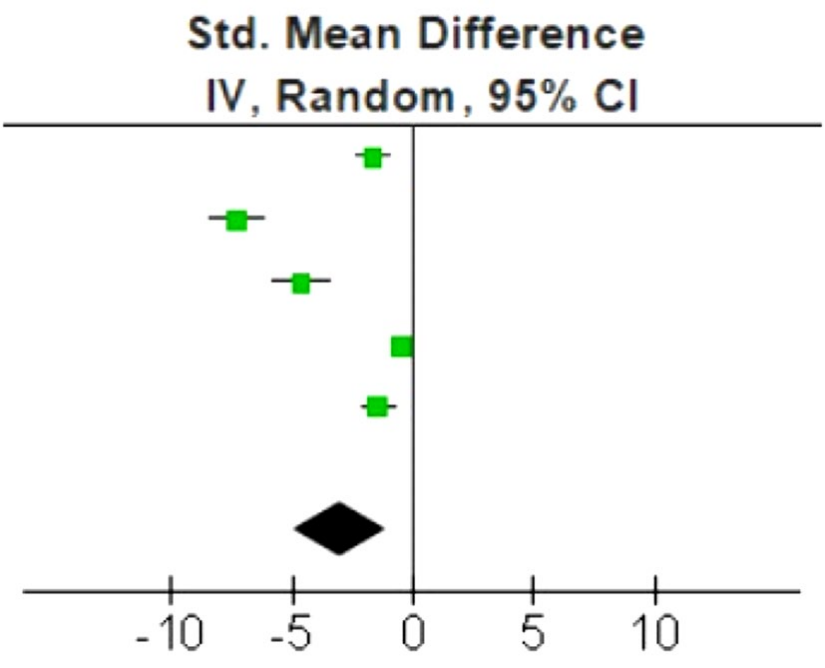
Forest plot with SMD of the five studies included in our meta-analysis

Five studies were included with a total of 168 subjects (158 pathologic PFs and 134 healthy PFs) with mean age 46 ± 6 years [18–22]. No significant publication bias was detected. Studies were highly heterogeneous (*p* < 0.00001; *I*^2^ = 97%). Pathologic PFs showed significantly lower stiffness, with an SMD of -3.00 m/s (95% confidence interval: − 4.95 to − 1.06, *p* = 0.002), compared to healthy PFs. The SMDs of the corresponding studies are symmetrically presented in the forest plot (Fig. 1).

In all subjects, a longitudinal US scan of the PF was adopted to calculate the stiffness. Inter- and intra-reader reproducibility was not measured in any studies. Three of these works were cross-sectional studies, while the remaining two were longitudinal studies. One prospective case–control longitudinal study was performed before and after treatment of plantar fasciitis with extracorporeal shockwaves, reporting a significant increase in SWE velocity from baseline (3.8 m/s [1.5–5.1]) to the follow-up visit performed 3 months after treatment (5.2 m/s [4.55–6.74], *p* = 0.003) [18]. The other prospective longitudinal case–control study investigated the impact of physical therapy on clinical, B-mode, and SWE findings of patients with plantar fasciitis; the authors found that 3 months of physical therapy treatment relieved symptoms, with a significant increase of PF stiffness from baseline (59.6 ± 43.3 kPa) to follow-up (102.5 ± 47.2 kPa; *p* < 0.001), without significant changes of B-mode features [21]. All data from the meta-analysis are resumed in Table 1.

**Table 1:** Data from five studies included in our meta-analysis

Study	Fascitis			Normal			Weight (%)	Std. Mean difference
	Mean	SD	Total PF	Mean	SD	Total PF		IV, Random, 95% CI
Alviti 2019	3.34 m/s	1.23	20	5.13 m/s	0.89	20	20.3	− 1.63 [− 2.36, − 0.91]
Beydogan, 2021	2.43 m/s	0.27	45	4.78 m/s	0.37	40	19.4	− 7.24 [− 8.43, − 6.05]
Gatz 1, 2020	31.9 kPa	16.2	31	124.1 kPa	28.5	10	19.2	− 4.58 [− 5.85, − 3.32]
Gatz 2, 2020	59.57 kPa	43.3	43	82.23 kPa	47.3	43	20.7	− 0.50 [− 0.92, − 0.07]
Schillizzi, 2020	3.5 m/s	1.01	19	5.04 m/s	1.14	21	20.4	− 1.40 [− 2.10, − 0.70]
Total (95% CI)			158			134	100.0	− 3.00 [− 4.95, − 1.06]
Heterogeneity: Tau^2^ = 4.70; Chi^2^ = 132.92, df = 4 (*P* < 0.00001); *I*^2^ = 97%								
Test for overall effect: *Z* = 3.03 (*P* = 0.002)								

Note – Gatz 1 refers to [20], Gatz 2 refers to [21],

## Discussion

Our main finding is that patients with plantar fasciitis show decreased SWE velocity, with pathologic PFs being softer than healthy PFs.

Over the last few years, sonoelastography has been increasingly used as an imaging tool able to help in evaluating PF status and composition from both a quantitative and qualitative point of view, in addition to the conventional B-mode ultrasound imaging [23]. Two sonoelastography methods are commonly used in musculoskeletal research and clinical practice: strain elastography, in which a mechanical force compresses the tissues axially, and SWE, in which compressive acoustic waves dynamically provide local stress in the soft tissues [24]. Strain elastography enables one to assess the deformation of the soft tissues along the propagation axis of the beam through the analysis of the RF signal along each line of scanning. The resulting colour elastogram that is generated is overlaid on the B-mode greyscale image, providing the operator with qualitative information about the tissue’s elasticity. The stiffness of PF may therefore be evaluated only qualitatively, although pseudo-quantitative information can be obtained by calculating strain ratios, which can be used to compare the PF strain with that of closing healthy tissue.

SWE allows a quantitative and reproducible approach for evaluating PF stiffness, being less operator-dependent than SES [25]. A focused acoustic radiation force is delivered from a linear US probe to induce shear waves throughout the soft tissues. These shear waves propagate perpendicularly at a slower velocity than the US beam, resulting in particle displacements that can be calculated using a speckle tracking algorithm. Tissue displacement maps are used to measure SWE velocity, expressed in meters per second. The distribution of shear wave velocities at each pixel is directly related to the shear modulus G (ratio of stress to strain), which is calculated by a simple mathematical equation and expresses the tissue stiffness and elasticity in units of pressure, usually kilopascals. In contrast to strain elastography, SWE allows quantitative measurements from any portion of the investigated PF within the colour elastogram due to the sequencing of particle displacements made possible by ultrafast analysis [23]. According to published studies on plantar fasciitis, SWE seems to be able to identify those degenerative changes that involve the PF, including collagen breakdown and disorientation, matrix degradation, increased mucoid content, and angiofibroblastic hyperplasia, which lead to the softer appearance of the PF in SWE [1]. It is true that strain elastography may provide an immediate assessment of PF stiffness, but SWE is more reliable and less operator-dependent, providing “numbers” that might be used in clinical practice to detect PF changes due to plantar fasciitis and to identify any response to treatment.

However, although it might be stated that pathologic PFs have lower SWE velocities than healthy ones, data retrieved from the literature are highly heterogeneous; thus, based on published data, it is not possible to provide standardised cut-off values. The remarkable differences noted when comparing the published studies may be justified by differences in the estimation of SWE velocity between systems and according to the depth of the PF [26]. Further, SWE examination can be affected by the transducer pressure and angle, the use of a spacer may impact the assessment of SWE velocity, and the shear modulus depends on the orientation of the probe relative to the PF; therefore, different methods may have affected the results of previous studies [27, 28]. These are important issues that must be addressed to make this tool robust and accurate enough to be applied routinely. As a matter of fact, there are international recommendations that, based on strong evidence, suggest the use of elastography on lateral epicondylitis and Achilles tendinopathy [4], but there is still no strong evidence to support its use over other imaging techniques for the PF in clinical practice, given that the additional value over conventional B-mode imaging still needs to be demonstrated.

Some limitations of this study should be pointed out. First, only a relatively low number of studies met our inclusion criteria, highlighting the importance of further studies to better clarify the role of SWE in this setting. Then, few studies have investigated its diagnostic performance by reporting clear cut-off values, which still have not been standardised. Last, most are cross-sectional studies, so more longitudinal studies are required to understand how SWE might be applied in patients’ management.

In conclusion, interesting data have been published about the use of SWE on plantar fasciitis, and it seems to be able to distinguish pathologic from healthy PF, with the former presenting a softer appearance and lower SWE velocity. Promising results have also been reported in the evaluation of the treatment response to conservative therapies. Nevertheless, the results of published studies are strongly heterogeneous and require further investigation.

## Data Availability

The datasets generated during and/or analysed during the current study are available from the corresponding author on reasonable request.
